# Phenotyping of Chronic Obstructive Pulmonary Disease Based on the Integration of Metabolomes and Clinical Characteristics

**DOI:** 10.3390/ijms19030666

**Published:** 2018-02-27

**Authors:** Kalle Kilk, Argo Aug, Aigar Ottas, Ursel Soomets, Siiri Altraja, Alan Altraja

**Affiliations:** 1 Department of Biochemistry, Institute of Biomedicine and Translational Medicine, University of Tartu, Ravila 19, 50411 Tartu, Estonia; argo.aug@haigekassa.ee (A.A.); aigar.ottas@ut.ee (A.O.); ursel.soomets@ut.ee (U.S.); siiri@ebc.ee (S.A.); 2 Centre of Excellence for Genomics and Translational Medicine, Riia 23b, 51010 Tartu, Estonia; 3 Estonian Health Insurance Fund, Lastekodu 48, 10144 Tallinn, Estonia; 4 Department of Pulmonology, University of Tartu, Puusepa 8, 51014 Tartu, Estonia; alan.altraja@ut.ee; 5 Lung Clinic, Tartu University Hospital, Puusepa 8, 51014 Tartu, Estonia

**Keywords:** chronic obstructive pulmonary disease, metabolomics, exhaled breath condensate, phenotyping, GOLD stratification, sphingomyelin

## Abstract

Apart from the refined management-oriented clinical stratification of chronic obstructive pulmonary disease (COPD), the molecular pathologies behind this highly prevalent disease have remained obscure. The aim of this study was the characterization of patients with COPD, based on the metabolomic profiling of peripheral blood and exhaled breath condensate (EBC) within the context of defined clinical and demographic variables. Mass-spectrometry-based targeted analysis of serum metabolites (mainly amino acids and lipid species), untargeted profiles of serum and EBC of patients with COPD of different clinical characteristics (*n* = 25) and control individuals (*n* = 21) were performed. From the combined clinical/demographic and metabolomics data, associations between clinical/demographic and metabolic parameters were searched and a de novo phenotyping for COPD was attempted. Adjoining the clinical parameters, sphingomyelins were the best to differentiate COPD patients from controls. Unsaturated fatty acid-containing lipids, ornithine metabolism and plasma protein composition-associated signals from the untargeted analysis differentiated the Global Initiative for COPD (GOLD) categories. Hierarchical clustering did not reveal a clinical-metabolomic stratification superior to the strata set by the GOLD consensus. We conclude that while metabolomics approaches are good for finding biomarkers and clarifying the mechanism of the disease, there are no distinct co-variate independent clinical-metabolic phenotypes.

## 1. Introduction

Chronic obstructive pulmonary disease (COPD) is a common disease affecting >10% of the population over 40 years of age [1]. Due to its increasing prevalence [2] to become the third-leading cause of death worldwide by the year 2030 [2,3], it represents an escalating global health burden [4,5]. The progressive airflow obstruction in the conducting airways, marked by a decline in the forced expiratory flow in one second (FEV_1_) [6] and related dynamic hyperinflation [7], has formed the historical staging basis for COPD [8]. However, since COPD is a complex, heterogeneous condition [9], FEV_1_ appeared unable to describe in a clinically meaningful way the impact of COPD on the patient, exerted by symptoms, systemic effects [10], and risks of exacerbations and mortality [11]. Therefore, from 2011 onward, a larger view increasingly driven by respiratory symptoms and exacerbation risks was introduced by the Global Initiative for COPD (GOLD) to stratify patients with COPD into 4 categories (A–D) for more refined management [5,12]. Furthermore, with the precision medicine perspective and a shift towards a more personalized approach to pharmacotherapy [13], fairly advantageous phenotypes of COPD were created on the basis of clinical, physiological, radiological, biological, and genetic input data [9,14,15,16,17]. Nevertheless, none of these phenotypes is yet able to reflect the bio-clinical interdependence in COPD with sufficient certitude. Along with the trendy approach to the phenotype-directed new therapies, a tremendous interest exists towards the mechanisms [18], as well as to the biomarker-driven early diagnosis [19] and detection of the progression of COPD [20]. Metabolomics approach that focuses on metabolism encompassing the variety of metabolites and regulation of metabolic networks would be a key to reach these goals [21]. The strength of metabolomics, compared to genomics, transcriptomics, and proteomics, relies on the fact that metabolomes represent the status of a biological system in the closest fashion, as they result from the interaction of the genome with its environment, include compounds of non-genetic origin, and are integrated in the regulatory biosystem [22]. 

**Figure 1:**
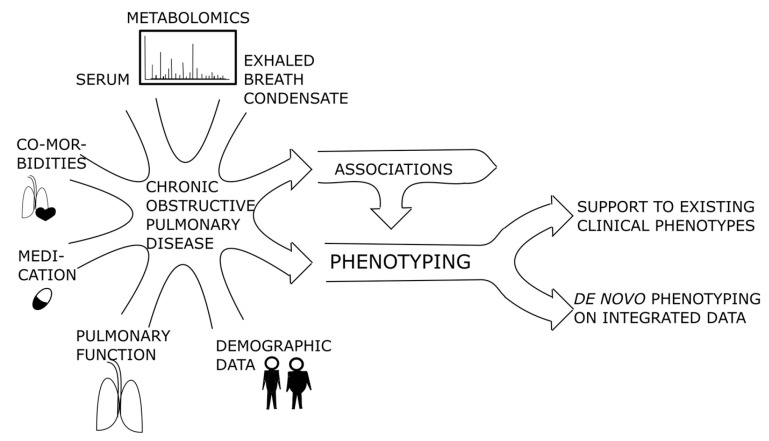
The principal flowchart of the study on integrated use of non-metabolomic variables and metabolomics-based biomarkers to phenotype COPD.

In COPD, beyond the biological accuracy of the analyses, non-invasiveness of the sampling is also critical. With this regard, analyses on both volatile [20] and non-volatile constituents [23] of exhaled breath condensate (EBC) have been proven increasingly useful in COPD research [20,24]. Metabolites account for a major part of the substances in EBC [25,26], as well as in the peripheral blood [27], sufficient to perform a metabolomics-based profiling of COPD. Nevertheless, although the metabolomic profiles have appeared applicable to distinguish between COPD and various other respiratory diseases [26,28], they have never been applied to recognize COPD phenotypes with simultaneous integration of a wide range of clinically identifiable markers that have been used over time for staging, assessment, and management of COPD, such as patients’ demographics, symptoms, pulmonary function, risks’ determinants, systemic markers, comorbidities, and management issues. Therefore, in this pilot study, we aimed to clarify whether a unique approach with integrating: (1) clinical and (2) demographic data with all metabolites from (3) EBC and (4) peripheral blood revealed by non-targeted approach, and (5) measurement results of pre-defined metabolites in peripheral blood could serve as a basis for COPD phenotypes (Figure 1). Since no studies with that many input variables have been done previously, we first aimed to determine how the clinical and metabolic parameters are associated to each other. Additionally, we tested the hypothesis that patients with stable COPD can be grouped into novel bio-clinical phenotypes without an a priori premise with synchronized use of non-metabolomic variables and metabolomics-centred biomarker signatures. 

## 2. Results

Twenty-five COPD patients and 21 controls were enrolled (Table 1). Characteristics related solely to the COPD patients are presented in Table 2. 

### 2.1. Association Studies

First, it was clarified, which metabolites, characteristics, or their composite signatures differentiate COPD patients from controls.

**Figure 2:**
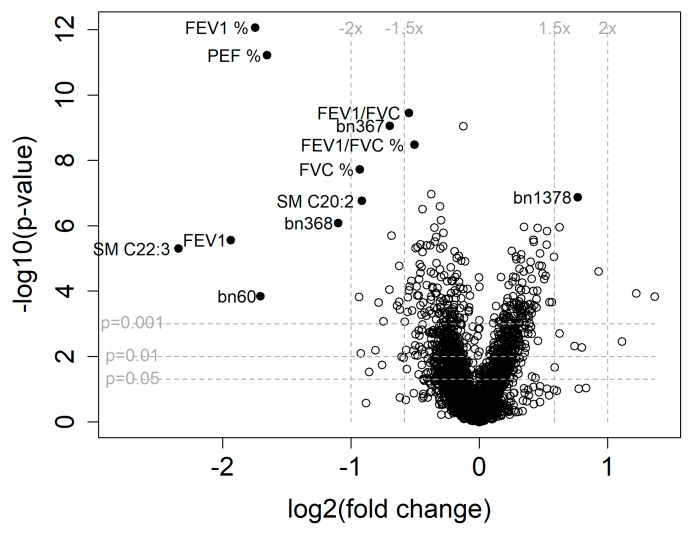
Volcano plot of the fold change (*x*-axis) vs. the significance of the change (*y*-axis) in the individual parameters between the patients with chronic obstructive pulmonary disease (COPD) and the control individuals. All clinical and demographic data and metabolic profile of exhaled breath condensate and serum including targeted analyses of the metabolites in serum were included except the dichotomous variable of smoking history (statuses of current or ex-smoker). Positive change indicates higher concentration or value for the patients with COPD. The most significantly different parameters, metabolites, and *m*/*z* values from metabolite profiles are annotated. Bn—signals from serum metabolic profile in negative ionization mode followed by the mass and charge ratio value; FEV_1_, forced expiratory volume in one second; FVC, forced expiratory volume; SM—sphingomyelin, followed by hydrocarbon chain length and number of double bonds. % indicated percent predicted for FEV_1_, FVC, and FEV_1_/FVC.

In direct comparison between the COPD and control groups with *t*-test, FEV_1_ and peak expiratory flow (PEF) displayed the most significant and pronounced differences. However, sphingomyelins (SM) with C20:2 and C22:3 fatty acid residues, as well as the *m*/*z* values 327, 367, and 368 in negative ionization serum profile in demonstrated differences comparable to those shown by the pulmonary function parameters, including the COPD-defining ones like the FEV_1_/forced vital capacity (FVC) ratio, in terms of their impact (Figure 2). The levels of all detected SM and hydroxylated SM (SM(OH)) were significantly decreased in patients with COPD (Table S2). The phosphatidylcholines (PCs) were more heterogeneous with regard to differentiating the COPD patients from controls. First, the level of PCs was generally decreased in patients with COPD, however, statistically significant were only the differences for polyunsaturated fatty acid (PUFA)-containing PCs with summary acyl-residue lengths between C36 and C40. LysoPCs showed a tendency towards accumulation in COPD patients. A sensitivity analysis was performed to evaluate the possible gender bias in control and COPD groups. With female samples excluded from the original 1167 statistically changed (*p* < 0.05) characteristics 793 (68%) remained statistically significantly changed. Among targeted metabolites only palmitoyl-lysoPC lost its significance from the original 33 statistically significantly altered metabolites. 

**Figure 3:**
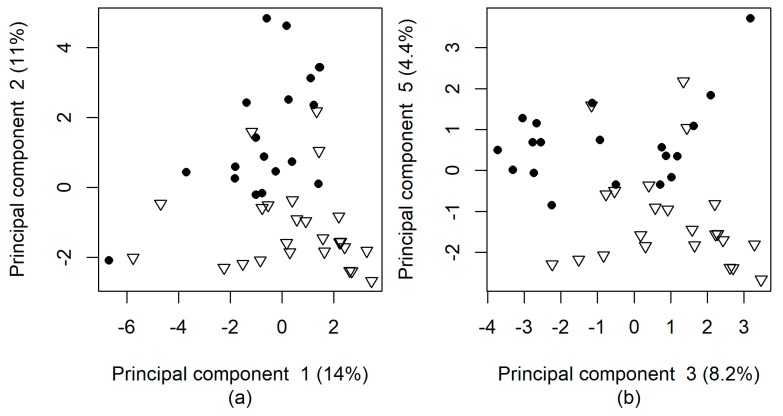
Principal component analysis of clinical and demographic data and metabolic profile of exhaled breath condensate and serum (including targeted analyses of the metabolites in serum) of the patients with chronic obstructive pulmonary disease (COPD, empty triangles) and the control individuals (solid circles). The percentage in parenthesis indicates the fraction from total variance explained by the respective principal component. (**a**) Principal components 1 and 2; (**b**) principal components 3 and 5.

Principal component analysis (PCA) searches for variance patterns in data matrices in an unsupervised manner i.e., without prior classification of samples. The first principal component (i.e., the highest variance) from whole data was associated mainly with data from serum metabolic profiles (Table 3) and was incompetent to differentiate COPD patients from controls (Figure 3a). Principal components 2, 3, and 5 were capable of partly separating the patients and controls (Figure 3b). These principal components were combinations of clinical parameters, sphingolipids, and PCs from the targeted metabolomics and several distinct *m*/*z* values from the untargeted metabolic profile (Figure 2, Table S1). Presence of smoking history was the most influential individual parameter for components 2, 3, and 5, but omission of it from the input data did not produce a restructuring in the principal components. Analysis performed solely on the metabolic profiles of EBC was not able to separate the COPD patients from controls on the basis of any principal component (Figure S1). On the other hand, a separation was achieved if either only the serum metabolic profiles or the data of the targeted analysis of metabolites in the serum were used.

Next, it was evaluated, which of the COPD clinical parameters, treatment regimens, or co-morbidities associate with which metabolites or signals in the metabolic profiles. Correlations between the clinical/demographic parameters and metabolomics data were calculated (Table 4). Smoking and COPD related clinical parameters were associated with various lipid species. Lipids also correlated with body mass (Table S4), but the species having the strongest associations were different. Body mass was also associated with the glutamate metabolism in a similar pattern, as did heart failure and emphysema. Calculations of associations with the use of long-acting beta_2_-agonists were omitted since these drugs were in use in all but one patient. Treatment regimens containing inhaled glucocorticoids, irrespective of other concomitant drugs, altered PC and SM species, as well as the total SM levels. However, while the smoking pack-years correlated more with polyunsaturated lipids, the glucocorticoid use was notably associated with saturated lipids (Table 4). The associations between the use of long-acting muscarinic antagonists and theophylline and the quantified metabolites were inferior to that of the inhaled glucocorticosteroids. Sensitivity analyses performed with exclusion of the signals suspected to originate from the drugs themselves, revealed no dependence of the metabolic profiles of either blood or EBC on the medication.

### 2.2. Global Initiative for COPD (GOLD) Strata Characterization

**Figure 4:**
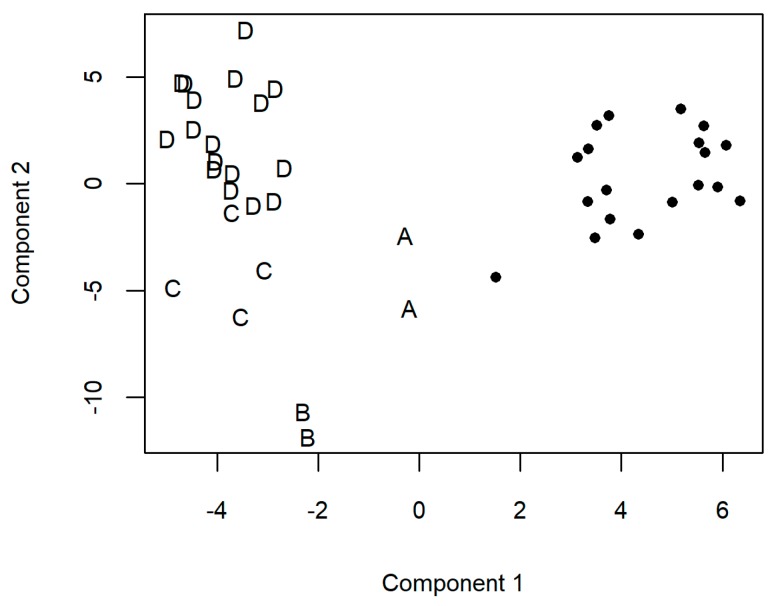
Sparse partial least squares discriminant analysis (sPLSDA), based on clinical and demographic data and metabolic profile of exhaled breath condensate and serum including targeted analyses of the metabolites in serum from the patients with chronic obstructive pulmonary disease (COPD) and the control individuals (solid circles) enrolled for the integrated metabolomics-clinical/demographic phenotyping analyses. A–D, patients with COPD at GOLD (The Global Initiative for COPD) stages A–D [5].

Sparse partial least square discriminant analysis (sPLSDA) on the whole data were used to further specify, which parameters or patterns define COPD in general and which could be associated with the sub-classes of COPD defined by GOLD [5]. The first component in sPLSDA successfully grouped controls separately from COPD patients and placed the GOLD A patients closer to the controls than to the GOLD B-D patients (Figure 4, Figure S2). The first component was built from clinical parameters, SM(OH) C22:1, SM C24:0, and SM C22:3, and serum profile signals, similarly to the results obtained with the *t*-test. The second component was mostly dependent on the metabolic profiles of serum in the negative ionization with *m*/*z* > 800 and split COPD categories B, C, and D from each other. 

ANOVA and subsequent comparison with the Tukey HSD test applied on the GOLD categories A–D revealed significant (*p* < 0.0001) differences between the strata of COPD for FEV_1_, FVC, PEF, exacerbations, and the *m*/*z* values 1069 and 1317 in the negative and the *m*/*z* value 259 in the positive ionization of the non-targeted analyses of the serum metabolic profiles. The latter *m*/*z* was high in the GOLD A COPD, whereas the *m*/*z* 1069 and 1317 were high in the GOLD B COPD. Lower significance (with *p* values being between 0.05–0.0001) was assigned to 427 different *m*/*z* values in the metabolic profiles, lysoPC with acyl residues C16:0, C17:0, C18:0, C18:1, and C18:2 (being high in GOLD A COPD), kynurenine (high in the GOLD B COPD), putrescine ratio to ornithine (high in GOLD C COPD), and ornithine ratio to serine (low in GOLD C COPD). 

### 2.3. Classification of Chronic Obstructive Pulmonary Disease (COPD) with Integrated Use of Non-Metabolomic Variables and Metabolomics-Based Biomarkers

**Figure 5:**
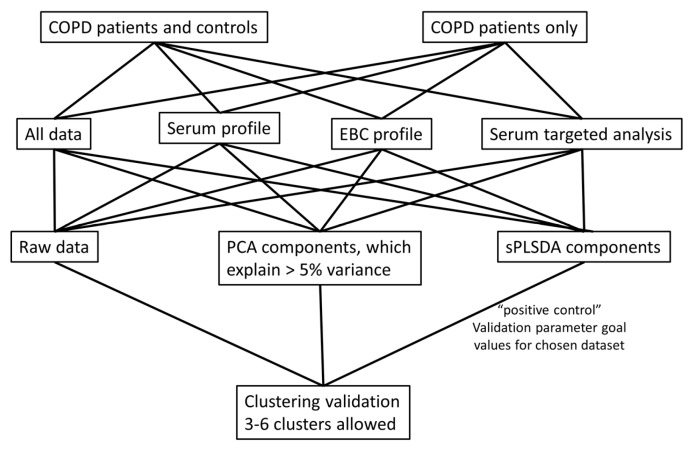
The flowchart of de novo phenotyping on integrated use of non-metabolomic variables and metabolomics-based biomarkers to phenotype COPD. COPD, chronic obstructive pulmonary disease; EBC, exhaled breath condensate; GOLD, global initiative for COPD; PCA, principal component analysis; sPLSDA, sparse partial least squares determinant analysis.

**Figure 6:**
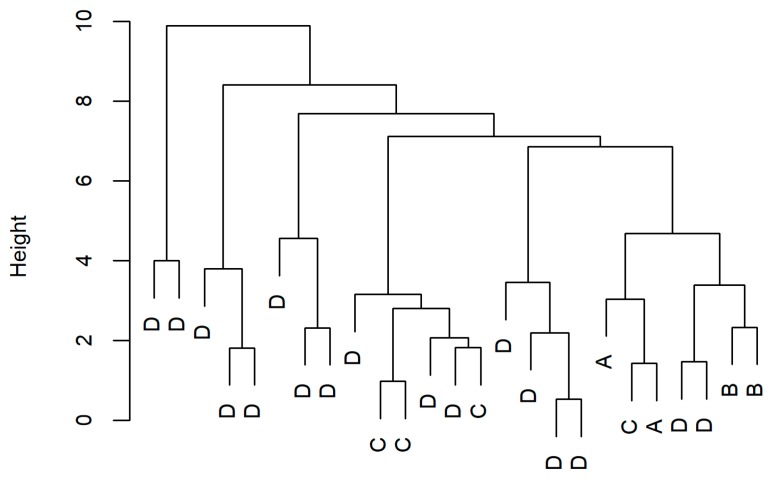
Hierarchical clustering of the patients with chronic obstructive pulmonary disease (COPD) (*n* = 25) enrolled for the integrated metabolomics-clinical/demographic phenotyping analyses, based on the 5 main principal components of the complete data. The letters A–D indicate how the individual patients with COPD at GOLD (The Global Initiative for COPD [5]) stages A–D, respectively, are allocated. The optimal number of subclasses/clusters by the Calinski-Harabasz psudo F-statistic was six.

To explore, whether the integrated analysis of the clinical and metabolic data classifies our patients with COPD without a premise of concordance with the clinical consensus-based stratifications [5,8], hierarchical cluster analysis was performed. The workflow for sub-classification of the COPD patients was as described in Figure 5. A total of 24 different sets of data were subjected to clustering. For quality assessment it was hypothesized that if a better clustering than the GOLD A–D exists, the quality parameters of this clustering would approach the values obtained with the GOLD A–D-based [5] sPLSDA clusters from the same dataset (i.e., the “positive control”). Comparisons for the quality characteristics did not yield a model comparable with the positive control. The best results were achieved if the control individuals were excluded and the five highest principal components from the complete data were used (Table S3, Figure 6). 

## 3. Discussion

In the current study, the clinical/demographic and metabolomic data were pooled into one integrated database and mined for both expected and novel sub-classes of COPD within. PCA on the COPD patients and control individuals indicated that the clinical parameters and targeted analysis of certain lipid species were the best to differentiate patients from controls. The metabolic profile of serum appeared to have high variance, which, on the other hand, had little correlation with the presence of COPD. The metabolic profile of EBC contributed to the principal components 3 and 5. The principal component 3 separated a subgroup of control individuals from other controls and COPD patients suggesting that the respective data could be used to test for the absence of COPD rather than for its presence. Former trials on the suitability of EBC for the diagnostics of COPD or monitoring the course of the disease have remained controversial [29]. Although contributing to the principal components 3 and 5 in our current study, the metabolic profiles of EBC alone were not able to differentiate patients from controls. Therefore, the value of metabolic profiles of EBC for COPD diagnosis remains questionable. It should be kept in mind, however, that the low efficacy of principal components does not necessarily mean that an individual metabolic signal cannot be specific for COPD. Furthermore, low resolution in untargeted analysis may diminish the significance of a specific marker due to being binned together with other compounds with similar *m*/*z* value. In the context of question whether there exists a COPD specific profile of metabolites, it is noteworthy to realize that the controls and COPD patients tend to overlap for all principal components despite the fact that they are not fully matched in age, sex or medical treatment. The latter is a major limitation of the current study, although its effects could be ameliorated by adjustment of the rest of the results for the gender and age data. Although gender is expected to influence some parameters (e.g., creatinine or steroid hormones) PCA results (Table S1) indicate that gender is associated with major variation in the whole integrated data. ANOVA sensitivity tests with females excluded, demonstrated some sensitivity in untargeted metabolic profiles (ca. 66% remaining unaltered), but only minimal sensitivity in other characteristics. 

sPLSDA, on the contrary to PCA, searches for the highest variations between pre-determined groups. In the current study, unlike PCA, sPLSDA was able to separate the different GOLD stages of COPD. The first dimension differentiated controls and GOLD A COPD from others, based on a combination of clinical/demographic and metabolic parameters. Separation of the GOLD stages B–D in the second dimension was mainly owing to the *m*/*z* values over 800 Da in the metabolic profile of serum in the negative ionization mode. The biomolecules behind these signals are most likely proteins and glycolipids, since (1) just very few other potentially matching metabolites have so high molecular weight and (2) other biopolymers (e.g., nucleic acids) are unlikely to be present in high enough amounts. Turnover of protein during the lung tissue remodelling in COPD, stress of the endoplasmic reticulum, and cellular response to unfolded proteins [30] are the likely mechanisms for this kind of changes in the protein profile. Remarkable changes in glycolipid profile have less supporting data, nevertheless, it cannot be excluded, when involvement of various other lipids, as discussed below, is considered. 

The cornerstone of the diagnosis of COPD is measurement of the post-bronchodilator pulmonary function by spirometry [5]. Hence, the impact of parameters like FEV_1_, PEF, and FVC in separating the patients with COPD from controls was expectedly high. Emergence of smoking history as the top contributor was also not surprising, as the linkage between smoking and COPD has been recognized for a long time [31]. When smoking history was removed from the input parameters, the remaining principal components still maintained their primary composition indicating that other indices, including the metabolic biomarkers, were sufficient for the currently observed separation of COPD patients from control individuals. Next to the clinical parameters, from the metabolic signals, SMs and, to a lesser extent, PCs, allowed the differentiation of COPD patients from controls. The role of acylcarnitines was marginal compared to other lipid species measured. sPLSDA selected SM(OH) C22:1, SM C24:0, and SM C22:3 along with the clinical/demographic parameters to differentiate the controls and patients with GOLD A COPD from the rest of the COPD patients. *t*-Test highlighted SM C20:2 and SM C22:3, along with some unidentified signals from the untargeted metabolic profile, as indicators comparable to the spirometry variables for their impact. A recent sphingolipid analysis assigned specific metabolic pathways with either emphysematous or frequent exacerbator phenotypes of COPD [32]. Our present results are in agreement that the history of exacerbations is associated with sphingolipids, although the sphingolipid species quantified were different in both studies. In our study, particularly the SM(OH) and unsaturated C18 fatty acid residues had the strongest associations with both exacerbations and emphysema (Table 4). 

Lipids exert multiple roles in the body and many of them are potentially relevant for the pathogenesis of COPD. Different lipid species are in a dynamic balance with each other, further obscuring their precise relation with the pathogenesis. Our observation of decrease in certain lipid levels and accumulation of lysolipids in blood indicate a dominance of lipid catabolism in COPD. In the relevant cell or animal models [33,34], it has been suggested that fatty acid residues that can be harvested from lipids could serve as energetic substrates in the conditions of inhibited glycolysis and up-regulated β-oxidation. On the other hand, this concerns more triglycerides and carnitine esters than sphingo- and phospholipids. Hence, we consider this sort of shift in the utilization of energetic substrates unlikely to be the major contributor for the lipid changes currently observed in COPD. Inflammatory and immune regulations use eicosanoid type lipid mediators derived from polyunsaturated fatty acids (PUFAs) and arachidonic acid (C20:4) in particular. Phospholipids serve as a reserve for PUFAs [35] and the current results partly fit a model, where PUFAs are continuously utilized in COPD and its exacerbations to satisfy the further increase in the demand for arachidonic acid. The remarkable association of sphingolipids with COPD points towards a specific role of these lipids in the pathogenesis of COPD. Indeed, in addition to eicosanoids, sphingolipids can specifically modulate inflammation and immune response [36,37]. Sphingolipids have been shown to correlate with sputum granulocytes [38]. Yet another role for phospholipids is them being surfactant in the lungs. The surfactant level may be a key factor in the development of emphysema [33,39,40]. While PCs being the largest contributors to surfactant, their level may relate to COPD subtypes, as currently observed. Taken together, eicosanoid- and sphingolipid-mediated immune/inflammation regulation and changes in surfactant composition may all contribute to the development and progression COPD. 

Amino acids, another large group of metabolites measured by targeted approach, were inferior to the lipids or clinical parameters in terms of separation or sub-classification of COPD. Nevertheless, particularly in the principal component 5, arginine- and glutamine-related amino acids (arginine, citrulline, asymmetric dimethylarginine and glutamine) and the ornithine/serine ratio ranked high in the factor loadings (Table S1). An earlier study built on initial metabolic profiling on NMR spectrometry identified amino acids as a major hallmark of COPD [41]. Still, NMR spectrometry may have on technical reasons favoured stronger and more specific signals from amino acids over lipids. In addition to study the amino acids in greater details, significant decreases in PUFAs and lipoproteins were also reported. This [41] and another report from the same authors [42] highlight the increased glutamine, aspartate, arginine, phenylalanine, and branched-chain amino acid levels in patients with COPD. With the exception of valine from the set of branched chain amino acids, the increased level of these amino acids was confirmed also by our current results. We were, however, not able to confirm the suggested correlations of certain amino acids with the severity of COPD or the presence of emphysema. The reason could be that the proportion of mild COPD in our study was too limited for a sufficient statistical power or, as described also in the studies by Ubhi et al. [41,42], the level of these amino acids may depend more on other factors like the body mass index and inflammatory reaction than on the presence of COPD per se. Arginine pathways are important in the generation of nitric oxide, an important pathway impacting pathogenesis of both COPD and asthma [43,44]. In COPD, up-regulation of the arginine pathway has been linked to exacerbations [45], although its use as a biomarker is questionable [46]. In our analysis, the amino acid levels in serum did not have significant correlations with any of the particular COPD phenotypes. However, among the amino acids that contributed to the principal components that separated controls from COPD patients, arginine and glutamine indeed had higher contribution than the rest of the amino acids. In an isotope labelling study, no change in the amino acid levels in serum was observed, but yet the systemic arginine level in COPD was found to be increased [47]. Interestingly, no change in the production of nitric oxide was observed [47]. Polyamines, such as putrescine and spermidine, are also linked to the metabolism of arginine, glutamine, and most directly to that of ornithine. Their role in the body is multifaceted and includes cell proliferation, control of differentiation, and regulation of nitric oxide synthesis among others. We found no statistically significant differences for polyamines between COPD patients and controls. Instead, a subset of COPD patients with an increased level of putrescine over ornithine was found. Since the alterations in the metabolism of arginine and glutamine in COPD have been confirmed previously in several studies [41,47,48] and that nitric oxide has remained unreliable, the alternative pathway to polyamines may deserve more attention in the future studies. 

Clustering of COPD patients based on their metabolic profiles, targeted metabolite analyses, or combinations of metabolic and clinical data did neither lead to a good match with the GOLD classification nor a novel but well-defined grouping. This may be due to heterogeneity of the disease at the molecular level, but also the small size of the study group, which is one of the major limitations of the study. This limitation, however, is counterbalanced with the large number of metabolomics variables integrated with the clinical and demographic ones. Heterogeneity of COPD has origins both in genetic background and environmental factors. Out of the latter, drugs and co-morbidities were taken into account in this study, but none of the factors had as dominant effect on metabolome that it would have dictated the classification. For instance, SMs were the top COPD associated metabolites in this study and have previously been used for COPD phenotyping [32]. We found some SM species positively correlated with pack-years or presence of emphysema and others associated with medications used or body mass. The actual profile of SMs is therefore dependent on more than just COPD defining parameters. With no parameter being dominant, the SMs, or the metabolic profile in general, will be very individual. A personalized medicine approach might be possible if all associated variables can be identified for a person, but defining distinct metabolic phenotypes seems unreasonable. 

## 4. Materials and Methods 

### 4.1. Study Individuals

The final study protocol was approved by the Ethics Committee on Human Research of the University of Tartu (approval 202T-10, 23 March 2011) and all procedures were conducted accordingly to the ethical standards of the World Medical Association Declaration of Helsinki: Ethical Principles for Medical Research Involving Human Subjects. All participants were recruited from the Department of Pulmonary Medicine, Tartu University Hospital, Tartu, and informed consent was obtained in written form from all individuals before entering the study. Patients diagnosed as having stable COPD of all categories and degrees of severity according to the Global Initiative for COPD consensus document [5] were included into the study. All patients with COPD were required to have their post-bronchodilator FEV_1_/FVC ratio below 0.7. Patients with a current exacerbation of COPD or an active malignancy were excluded. The patients with COPD were treated accordingly to the group of their disease (A–D) in concordance with the GOLD consensus report [5]. Healthy non-allergic never-smokers with the pulmonary function within their normal range were incorporated for reference. All study individuals were required to be free of upper and lower respiratory tract infections (including acute bronchitis and bronchiolitis, exacerbations of chronic bronchitis and pneumonia) during the study and for 4 weeks before the entry into the study. Complete data, appropriate for either the COPD patients, control individuals or both, that included main demographic and anthropometric indices, variables characterizing smoking habits, symptoms according to the modified Medical Research Council (mMRC) dyspnoea scale [49], pulmonary function data including forced post-bronchodilator flow-volume spirometry and lung diffusing capacity variables, COPD exacerbations during the previous 12 months, as well as the daily treatments for COPD and the presence of major concomitant diseases (heart failure, coronary heart disease, arterial hypertension, asthma, pulmonary hypertension, obstructive sleep apnoea, depression, diabetes, and osteoporosis), were prospectively collected and transferred from the official medical records to form the database for the study. The spirometry [50] and lung diffusing capacity [51] testing were performed in concordance with the American Thoracic Society/European Respiratory Society standards, whereas multi-ethnic [52] and Finnish reference values were used for spirometry [52] and diffusing capacity [53] parameters, respectively. A current smoker was defined as a person, who currently smoked ≥1 cigarette per day and an ex-smoker was defined as the one, who had quit smoking for more than 6 month prior to the study. Significant emphysema was defined as the presence of the proportion of the lung affected by emphysema (an attenuation below −950 Hounsfield units on multi-detector computed tomography imaging) of at least 10% [54]. The patients with COPD were categorized according to both the FEV_1_ level (by the GOLD 1–4 classification) [8] and the multidimensional GOLD A–D stratification [5]. 

### 4.2. Sample Collection and Processing

Samples of exhaled breath condensate (EBC) were collected from individuals using a single-use Rtube™ set (Respiratory Research, Inc., Austin, TX, USA) with the condenser precooled down to −80 °C by a cooling sleeve according to manufacturer’s instructions and published recommendations [29,55]. The patients breathed tidally through a mouthpiece with the nose closed using a nose-clip for 10 min. Intake of food or drink (except unflavoured drinking water) was disallowed for 3 h prior to the collection of EBC. The EBC samples at an amount of 1.5 ± 0.25 mL were divided into 500 µL aliquots and stored at −80 °C until used. For mass spectrometry, the EBC aliquots were thawed, 300 μL of ice-cold methanol was added to 100 μL of EBC, and the mixture was centrifuged for 15 min at 15,800× *g*.

Five mL of peripheral venous blood was collected using BD Vacutainer with clotting activator. The clot was centrifuged for 20 min at 1300× *g* within 1 h after collection. The serum was isolated and kept at −80 °C until analysis. For analyses, the serum samples were thawed, the proteins were precipitated with the addition of methanol up to 3:1, *v*:*v* and centrifuged at 15,800× *g* for 15 min. The supernatants were transferred to Eppendorf tubes and lyophilized for 18 hours. The dried samples were then dissolved in water and methanol at a 1:3 ratio (*v*:*v*) and allocated for mass spectrometry. 

### 4.3. Mass-Spectrometry Analysis

For untargeted analyses, the pre-prepared EBC and serum samples were randomized and analyzed on a QTRAP 3200 (Sciex, Framingham, MA, USA) mass spectrometer. Ten μL of redissolved serum and 50 μL of EBC was directly injected and analyzed in isocratic flow of 0.05 mL/min water, 0.15 mL/min methanol, and 0.1% formic acid. Electrospray ionization MS scans were performed in negative and positive modes with a rate of 1000 amu/s between the mass ranges of 90 to 1400 Da. Ionspray voltage, declustering potential, and entrance potential were set to 4500 V, 20 V, and 10 V, respectively with the use of corresponding negative voltages in the negative scanning mode. 

For targeted analysis, serum samples were measured on QTRAP 4500 (Sciex, Framingham, MA, USA), coupled to a high-performance liquid chromatography (HPLC) (Agilent 1260 series, Agilent Technologies, Waldbronn, Germany) using the AbsoluteIDQ p180 kit (Biocrates Life Sciences AG, Innsbruck, Austria) according to the manufacturer’s specifications. The resulting data were absolutely quantified concentrations of amino acids, acylcarnitines, biogenic amines, glycerophospholipids, hexose, and sphingolipids.

### 4.4. Data Analysis

The baseline data were compared between the control individuals and patients with COPD using Mann-Whitney *U*-test for continuous and Pearson’s chi-square test for categorical variables (Table 1). The obtained mass-to-charge ratios from the untargeted analysis were binned to 1 Da range with an in-house built Visual Basic based program. The binned mass spectra were analysed with R 3.3.0 (The R Foundation, Vienna, Austria). To compensate for batch variation, all measured samples were normalized to the total ion count. The intensities were logarithmically transformed. For integrated analysis of (1) the clinical and (2) demographic data with all metabolites from (3) EBC and (4) blood serum revealed by non-targeted approach and (5) measurement results of the pre-defined metabolites in blood serum, the data of all variables were transformed to reach values between 0 and 1 by the following means: the values from mass spectra from EBC and sera were allocated to logistic transformation (according to the formula 1/[(0.5 + e^−x^) × 2], whereas in case of the rest of the variables, the values were divided by the highest value in the respective variable. The data on mass spectra from EBC and sera were then combined with those on the targeted metabolomics analyses on sera, as well as on the clinical/demographic variables and the dataset was allocated for Student’s *t*-test, parametric analysis of variance (ANOVA) with Tukey HSD post hoc test, PCA, sPLSDA, and hierarchical clustering. In the latter, euclidean distance calculation with complete linkage agglomeration method were used. Sensitivity analysis with omission of samples with potential bias causing characteristics was used to confirm that the main findings hold if input data varies. sPLSDA (package mixOmics [56]) was used to identify the variables most responsible for allocation of the patients into GOLD strata [5] on one hand and as a positive control for clustering on the other hand. sPLSDA was done with 10 times repeated 5-fold cross-validation. In tuning 2 component model with 60 input parameters kept was chosen as the most optimal. Hierarchical clustering was also applied to the original dataset or PCA components, which explained at least 5% of the total variance. For the validation of the hierarchical clustering outcome, the packages clValid [57], clusterSim [58], and fpc [59] for R were used. The validation included the comparison of Calinski-Harabasz pseudo F-statistic, Davies-Bouldin index, Baker and Hubert adaption of Goodman and Kruskal’s gamma statistics, Hubert and Levine internal cluster quality index, Rousseeuw’s Silhouette internal cluster quality index, Dunn index, average proportion of non-overlap, entropy of the distribution of cluster memberships, and average and maximal values of within and between cluster distances. Several indexes were calculated with more than one package, depending on their availability. In addition, a subjective visual examination of the clustering was done. If the control individuals were included in the data input, the clustering was expected to branch out COPD patients and controls at the highest levels and not to misclassify more than two COPD patients or control individuals. 

## 5. Conclusions

In conclusion, our current study implies that COPD causes alterations in the metabolism of sphingolipids that are, for their amplitude and significance, comparable with the weight of the changes observed in pulmonary function testing. Untargeted profiling suggests that the sphingolipids may not be the only metabolites with such significance. Additionally, changes involving PUFAs and glycerophospholipids occur in COPD. While there exists a COPD associated metabolic profile, clustering of metabolic data even together with clinical-demographic variables did not reveal molecular phenotypes of COPD with well-defined boundaries. 

## Figures and Tables

**Table 1:** Baseline characteristics of the patients with chronic obstructive pulmonary disease (COPD) (*n* = 25) and the control individuals (*n* = 21).

Characteristic	Patients with COPD	Healthy Controls	*p*-Value ^a^
Age, years	67, 58–72 (42–78)	37, 27–62 (23–74)	<0.001
Gender, M/F (number, %)	25/0 (100/0)	9/12 (43/57)	<0.001
BMI, kg/m^2^	26, 23–31 (19–46)	24, 23–26 (22–37)	0.53
FVC, L ^c^	2.0, 1.5–2.5 (1.0–5.1)	3.6, 3.1–4.5 (2.6–6.7)	<0.001
FVC, % predicted ^c^	47, 39–57 (29–83)	89, 86–109 (84–129)	<0.001
FEV_1_, L ^c^	1.0, 0.8–1.2 (0.5–2.7)	3.3, 2.5–3.6 (2.1–5.4)	<0.001
FEV_1_, % predicted ^c^	29, 22–39 (14–81)	92, 87–102 (83–122)	<0.001
FEV_1_ reversibility, L ^c^	0, 0–0.08 (0–0.40)	0.11, 0.05–0.25 (0–0.28)	0.032
FEV_1_ reversibility, % ^c^	0, 0–8.0 (0–17)	4.1, 2.1–5.8 (0–7.0)	0.55
PEF, L/min ^c^	149, 125–229 (85–466)	403, 347–535 (296–701)	<0.001
PEF, % predicted ^c^	27, 23–39 (14–80)	94, 81–102 (75–108)	<0.001
FEV_1_/FVC ^c^	0.49, 0.42–0.65 (0.35–0.81)	0.81, 0.80–0.83 (0.77–0.93)	<0.001
FEV_1_/FVC, % predicted ^c^	62, 51–77 (43–106)	100, 96–103 (93–107)	<0.001
DLco, mmol/(min·kPa)	4.9, 2.6–5.8 (1.9–8.4)	7.4, 6.1–7.7 (4.7–11.2)	0.13
DLco, % predicted	46, 30–63 (25–85)	81, 78–96 (76–104)	0.001
Kco, mmol/(min·kPa·L)	1.1, 0.6–1.3 (0.4–1.5)	1.5, 1.3–1.6 (1.1–1.7)	0.004
Kco, % predicted	77, 44–103 (28–119)	98, 89–102 (78–107)	0.191

BMI, body mass index; DLco, diffusing capacity of the lung for carbon monoxide; FEV_1_, forced expiratory volume in one second; FVC, forced expiratory volume; IQR, interquartile range; Kco, diffusing coefficient of the lung for carbon monoxide; PEF, peak expiratory flow. Data presented as median, IQR (limits) unless otherwise specified. ^a^ Compared between groups using Mann-Whitney U-test for continuous and Pearson’s chi-square test for categorical variables. ^c^ All spirometry data used in this study represent post-bronchodilator values.

**Table 2:** Characteristics related solely to the patients with COPD.

Characteristic	Patients with COPD
Smoking- and COPD-related variables	
Smoking, pack-years	40, 27-53 (0–84) ^a^
Status of current smoker	10 (40)
Status of ex-smoker	14 (56)
mMRC dyspnoea score	2, 2–3 (1–4) ^a^
No. of COPD exacerbations/last 12 months	2, 1–2 (0–2) ^a^
Patients with frequent exacerbations ^b^	15 (60)
No. of concomitant diseases	2, 1–3 (0–5) ^a^
GOLD stage, 1/2/3/4	1/3/7/14 (4.0/12/28/56)
GOLD stage, A/B/C/D	2/2/4/17 (8.0/8.0/16/68)
GOLD 2017 stage, A/B/C/D	3/4/3/15 (12/16/12/60)
Significant emphysema	13 (52)
Medications used for COPD	
Long-acting beta_2_-agonists	24 (96)
Long-acting anticholinergics	20 (80)
Inhaled glucocorticosteroids ^c^	20 (80)
Sustained-release theophylline	17 (68)
Long-term domiciliary oxygen	10 (40)
Concomitant diseases and complications of COPD	
Heart failure	12 (48)
Coronary heart disease	7 (28)
Arterial hypertension	11 (44)
Asthma	7 (28)
Pulmonary hypertension related to COPD	4 (16)
Obstructive sleep apnea	1 (4.0)
Diabetes	2 (8.0)
Osteoporosis	2 (8.0)
Depression	4 (16.0)

Data are presented as number (%) unless otherwise specified. ^a^ median, IQR (limits); GOLD, Global Initiative for Chronic Obstructive Lung Disease; IQR, interquartile range; mMRC, modified Medical Research Council. ^b^ Defined according to the GOLD consensus document [5,12]. ^c^ Inhaled glucocorticosteroids were always used together with long-acting beta_2_-agonists in fixed combination inhalers.

**Table 3:** The relative importance of input sources for the principal components.

	Principal Component 1	Principal Component 2	Principal Component 3	Principal Component 4	Principal Component 5
Clinical parameters	3%	26%	25%	7%	31%
Serum profile	82%	29%	37%	22%	27%
EBC profile	7%	12%	25%	9%	15%
Serum amino acids ^a^	2%	7%	6%	11%	10%
Serum lipids ^a^	6%	25%	7%	51%	17%

^a^ Summary contribution of the individual compounds measured with a Biocrates AbsoluteIDQ180 kit.

**Table 4:** Associations between the most important clinical and metabolomic variables of the patients with COPD.

Metabolite	Pack-Years	FEV_1_ (L)	PEF (L/min)	Current Smoking Status	Inhaled Glucocorticoids	Long Acting Mus-Carinic Antagonists	Theophylline	Pulmonary Hypertension	Heart Failure	Emphysema	Exacerbations
	Pearson’s *r* ^a^			*p*-Value of *t*-Test or ANOVA ^a^							
Carnitine C18:2	0.49	0.33	0.24	0.58	0.55	0.55	0.56	0.92	0.98	0.72	0.014
Glu	−0.05	0.16	0.15	0.98	0.35	0.77	0.56	0.22	0.006	0.003	0.18
His	−0.50	−0.10	−0.08	0.40	0.39	0.69	0.44	0.74	0.37	0.16	0.65
Lys	−0.31	−0.27	−0.29	0.95	0.52	0.36	0.13	0.87	0.91	0.009	0.65
Kynurenine	0.18	0.49	0.58	0.71	0.17	0.49	0.07	0.42	0.05	0.20	0.87
Putrescine	0.55	−0.16	−0.23	0.07	0.16	0.72	0.50	0.83	0.36	0.26	0.39
lysoPC a C17:0	−0.50	0.30	0.28	0.20	0.31	0.59	0.15	0.88	0.14	0.82	0.14
lysoPC a C26:1	0.50	−0.02	0.13	0.21	0.41	0.48	0.17	0.58	0.03	0.06	0.62
PC aa C34:2	−0.53	0.03	0.00	0.30	0.25	0.46	0.23	0.35	0.55	0.41	0.49
PC aa C36:3	−0.49	0.02	−0.03	0.57	0.39	0.57	0.32	0.28	0.81	0.68	0.74
PC aa C42:0	−0.17	−0.20	−0.14	0.12	0.009	0.35	0.81	0.87	0.42	0.29	0.16
PC aa C42:2	−0.13	−0.21	−0.13	0.19	0.007	0.55	0.75	0.79	0.78	0.18	0.43
PC ae C34:3	−0.17	−0.40	−0.38	0.41	0.91	0.84	0.029	0.72	0.004	0.011	0.75
PC ae C36:3	−0.33	−0.18	−0.19	0.16	0.72	0.97	0.26	0.27	0.007	0.016	0.79
PC ae C38:3	−0.53	−0.06	−0.09	0.21	0.57	0.90	0.23	0.31	0.36	0.37	0.28
SM C16:0	0.06	−0.36	−0.37	0.15	0.11	0.007	0.40	0.34	0.40	0.09	0.69
SM C18:0	−0.12	−0.35	−0.34	0.11	0.001	0.38	0.22	0.61	0.85	0.35	0.07
SM C24:0	−0.51	−0.36	−0.42	0.78	0.26	0.69	0.045	0.78	0.49	0.06	0.20
SM C24:1	0.08	−0.52	−0.54	0.97	0.03	0.27	0.27	0.54	0.61	0.17	0.47
Glu/Gln	0.01	0.21	0.21	0.96	0.39	0.85	0.64	0.07	0.001	0.000	0.71
Glutaminolysis	0.09	0.55	0.59	0.64	0.21	0.28	0.19	0.19	0.022	0.004	0.27
Kynurenine/Trp	0.20	0.41	0.50	0.49	0.06	0.39	0.15	0.20	0.06	0.19	0.39
Thr/Ser	−0.08	−0.08	0.00	0.67	0.006	0.62	0.52	0.57	0.36	0.72	0.36
tSM	−0.10	−0.44	−0.45	0.40	0.019	0.034	0.20	0.53	0.41	0.06	0.26
tSM-non OH	−0.03	−0.45	−0.46	0.10	0.015	0.032	0.24	0.43	0.46	0.08	0.37
tSM-OH	−0.42	−0.26	−0.26	0.15	0.51	0.13	0.14	0.96	0.41	0.040	0.044
tSM-OH/tSM-non OH	−0.55	0.07	0.05	0.27	0.67	0.52	0.49	0.73	0.55	0.21	0.10

^a^ Correlations of at least moderate strength (±0.49 for *p* = 0.001) and *p* < 0.05 are bolded. aa, two fatty acid residues bound to glycerol with ester bonds; ae, one fatty acid residue bound to glycerol with ester bond, one with ether bond; PC, phosphatidylcholine with a fatty acid residue of given length; SM, sphingomyelin with a fatty acid residue of given length; SM(OH), hydroxylated sphingomyeline with a fatty acid residue of given length; tSM, total sphingomyelin content; FEV_1_, forced expiratory volume in one second; PEF, peak expiratory flow.

## Supplementary Materials

Supplementary materials can be found at http://www.mdpi.com/1422-0067/19/3/666/s1.
