# Supplementary material for: Phenotyping of Chronic Obstructive Pulmonary Disease Based on the Integration of Metabolomes and Clinical Characteristics
Source: Int J Mol Sci. 2018 Feb 27;19(3):666. doi: 10.3390/ijms19030666 (PMC5877527; doi:10.3390/ijms19030666)
Supplement: Supplementary file 1 [file ijms-19-00666-s001.zip › ijms-S1.pdf]

Supplementary material

Table S1. Parameters of the patients with chronic obstructive pulmonary disease (n = 25) and the control individuals (n = 21) that contributed the most to the first 5 principal components if all data (clinical and demographic data and metabolic profile of exhaled breath condensate and serum including targeted analyses of the metabolites in serum) were subjected to principal component analysis. The percentage in parenthesis after principal component indicates the fraction from total variance explained by the respective principal component. .

Principal component 1 (14%)	Principal component 2 (12%)	Principal component 3 (8.2%)	Principal component 4 (4.8%)	Principal component 5 (4.5%)
bp341	Ever smoked	Ever smoked	Ever smoked	Ever smoked
bp342	PEF (% predicted)	bn250	PC aa C36:1	PEF (% predicted)
bp343	PEF (L/s)	PEF (L/s)	PC ae C34:0	bn137
bp365	FEV_1 (% predicted)	en101	PC ae C36:1	FEV1 (% predicted)
bp280	bn175	FEV_1 (L)	PC aa C34:1	PEF (L/s)
bp366	SM(OH) C22:1	bn155	PC ae C38:2	SM C22:3
bp319	SM C20:2	bn69	PC ae C38:1	FVC (% predicted)
bp367	SM C26:0	bn205	PC ae C34:2	bn315
bp344	PC aa C36:6	FEV_1 (% predicted)	PC aa C32:0	bn195
bp318	PC aa C36:5	FVC (L)	PC ae C36:3	FEV_1 (L)
bp339	Total SM(OH)	bn171	PC ae C34:3	FVC (L)
bp317	PC aa C34:4	bn480	PC with PUFA	C14.1
bp320	bn367	bn793	PC ae C32:2	bn262
bp281	PC aa C38:5	PEF (% predicted)	PC ae C38:3	bn291
bp360	FVC (% predicted)	bn797	PC aa C36:0	ep610
bp340	bn368	ep444	PC ae C36:0	lysoPC a C28:0
bp304	SM(OH) C22:2	ep59	PC aa C36:2	lysoPC a C26:1
bp368	SM(OH) C16:1	FVC (% predicted)	SM(OH) C16:1	lysoPC a C26:0
bp345	bn282	bn532	PC aa C34:3	Age (years)
bp305	PC ae C38:0	bn504	PC aa C32:3	ep684
bp361	SM C24:0	ep342	PC aa C40:5	PC aa C26:0
bn292	bn280	ep382	PC ae C36:2	bn180
bp315	SM(OH) C24:1	bp558	PC ae C40:1	Glutamine
Ever smoked	SM(OH) C14:1	ep443	Total SM(OH)	PC aa C24:0
bp369	bn137	bn315	PC ae C42:2	bn245
bp316	Age (years)	bp564	SM(OH) C14:1	bn377
bp293	FEV_1/FVC (% predicted)	en265	SM(OH) C22:1	Acetylcarnitine/ free carnitine
bp850	PC aa C42:5	en482	PC aa C28:1	ep611
bp758	bn1378	bn114	bp166	Ornithine/Serine
bp321	PC aa C38:6	en163	SM C26:0	LysoPC a C28:1
bp760	PC with PUFA	en117	PC ae C34:1	bn190

aa, two fatty acid residues bound to glycerol with ester bonds; ae, one fatty acid residue bound to glycerol with ester bond, one with ether bond; bn, bp, en, and ep, mass-to-charge (m/z) value obtained from the mass spectra from serum in negative ionization, from serum in positive ionization, exhaled breath condensate (EBC) in negative ionization and from EBC in positive ionization respectively; FEV_1, forced expiratory volume in one second; FVC, forced expiratory volume; PEF, peak expiratory flow; PC, phosphatidylcholine, PUFA, polyunsaturated fatty acids; SM, sphingomyelin.

Table S2. Concentrations (µM) of quantitatively measured metabolites in serum of control individuals and COPD patients with 2014 year A-D stratification. <LOQ below quantification limit.

Metabolite	COPD A	COPD B	COPD C	COPD D	Controls
Free carnitine	49.2 ± 30.3	34.9 ± 3.4	32.2 ± 11.5	32.6 ± 9.8	32.8 ± 7.8
AC C10 *	0.2 ± 0.3	0.2 ± 0	0.2 ± 0.2	0.1 ± 0.2	0.4 ± 0.4[d]
AC C10:1	<LOQ	<LOQ	<LOQ	<LOQ	<LOQ
AC C10:2	<LOQ	<LOQ	<LOQ	<LOQ	<LOQ
AC C12	<LOQ	<LOQ	<LOQ	<LOQ	<LOQ
AC C12:DC	<LOQ	<LOQ	<LOQ	<LOQ	<LOQ
AC C12:1	<LOQ	<LOQ	<LOQ	<LOQ	<LOQ
AC C14	<LOQ	<LOQ	<LOQ	<LOQ	<LOQ
AC C14:1	0.05 ± 0.06	0.03 ± 0.00	0.04 ± 0.03	0.03 ± 0.03	0.07 ± 0.05
AC C14:1 OH	0.01 ± 0.02	0.01 ± 0.01	0.00 ± 0.01	0.01 ± 0.01	0.02 ± 0.01
AC C14:2	0.02 ± 0.02	0.01 ± 0.01	0.01 ± 0.01	0.01 ± 0.01	0.02 ± 0.01
AC C14:2 OH	<LOQ	<LOQ	<LOQ	<LOQ	<LOQ
AC C16	<LOQ	<LOQ	<LOQ	<LOQ	<LOQ
AC C16 OH	<LOQ	<LOQ	<LOQ	<LOQ	<LOQ
AC C16:1 **	0.03 ± 0.04	0.01 ± 0.02	0.02 ± 0.02	0.01 ± 0.02	0.04 ± 0.01[d]
AC C16:1 OH	0.01 ± 0.01	0.01 ± 0.01	0.00 ± 0.01	0.00 ± 0.01	0.01 ± 0.00
AC C16:2	<LOQ	<LOQ	<LOQ	<LOQ	<LOQ
AC C16:2 OH	<LOQ	<LOQ	<LOQ	<LOQ	<LOQ
AC C18 *	0.06 ± 0.02	0.05 ± 0.00	0.04 ± 0.01	0.04 ± 0.01	0.06 ± 0.02[d]
AC C18:1	0.17 ± 0.13	0.12 ± 0.02	0.11 ± 0.01	0.11 ± 0.06	0.13 ± 0.05
AC C18:1 OH	<LOQ	<LOQ	<LOQ	<LOQ	<LOQ
AC C18:2	0.06 ± 0.03	0.05 ± 0.02	0.04 ± 0.01	0.04 ± 0.02	0.04 ± 0.01
AC C2	8.3 ± 6.5	4.3 ± 0.01	5.3 ± 2.7	5.3 ± 2.3	8.8 ± 4.8
AC C3	0.6 ± 0.04	0.4 ± 0.01	0.4 ± 0.1	0.5 ± 0.2	0.4 ± 0.1
AC C3 OH	<LOQ	<LOQ	<LOQ	<LOQ	<LOQ
AC C3:1	<LOQ	<LOQ	<LOQ	<LOQ	<LOQ
AC C4	<LOQ	<LOQ	<LOQ	<LOQ	<LOQ
AC C4:1	<LOQ	<LOQ	<LOQ	<LOQ	<LOQ
AC C5	<LOQ	<LOQ	<LOQ	<LOQ	<LOQ
AC C5 M DC	<LOQ	<LOQ	<LOQ	<LOQ	<LOQ
AC C5:1	<LOQ	<LOQ	<LOQ	<LOQ	<LOQ
AC C5:1 DC	<LOQ	<LOQ	<LOQ	<LOQ	<LOQ
AC C6:1	<LOQ	<LOQ	<LOQ	<LOQ	<LOQ
AC C7 DC	<LOQ	<LOQ	<LOQ	<LOQ	<LOQ
AC C8	<LOQ	<LOQ	<LOQ	<LOQ	<LOQ
AC C9	<LOQ	<LOQ	<LOQ	<LOQ	<LOQ
Ala *	605.5 ± 121	482.5 ± 42	404.3 ± 87	439 ± 136	377.9 ± 68[a]
Arg **	147.6 ± 84	83.2 ± 20.7	167 ± 34.5	132.5 ± 35.5	99.5 ± 27~~6.5~~[c,d]
Asn	58.1 ± 1.6	45.9 ± 3.5	63.3 ± 23.7	50.9 ± 14.8	50.3 ± 9
Asp	19.3 ± 2.1	21.5 ± 6.6	19.6 ± 11.2	18 ± 3.4	22.7 ± 7.1
Cit	40 ± 3.5	37.7 ± 23.2	32.7 ± 20.3	31.5 ± 13.6	29.7 ± 8.4
Gln ***	1066 ± 175[b]	671.5 ± 7.8[a]	906 ± 230	947 ± 164	689 ± 74[a,c,d]
Glu	54.5 ± 21.4	48.5 ± 1.1	35.6 ± 13.1	53.6 ± 20.9	53.5 ± 21.7
Gly	323.5 ± 21.9	247 ± 72.1	359 ± 170.3	268.5 ± 99	291.7 ± 117
His	95.1 ± 16.8	80.2 ± 10.2	89.9 ± 21.3	87.9 ± 13.2	87.9 ± 12.6

Ile	96.5 ± 37.5	75.6 ± 8.4	86.6 ± 18.6	92.9 ± 31.4	70.2 ± 16.8
Leu	180 ± 60.8	141 ± 4.2	149.5 ± 31.7	178.1 ± 79.3	137.9 ± 36
Lys	186.5 ± 16.3	160 ± 15.6	196.3 ± 34.9	189.6 ± 33.7	200 ± 37.8
Met	27.6 ± 8	20.3 ± 5.1	29.1 ± 7.3	24.4 ± 7.9	22.8 ± 4.5
Orn **	116.7 ± 25.9	109.3 ± 30.8	82.4 ± 23.4	113.4 ± 23.6	80.1 ± 22.4 [d]
Phe **	75.5 ± 4.2	78 ± 10	77.9 ± 8.3	76.2 ± 13.3	62.7 ± 10.8 [d]
Pro	355 ± 178	296.5 ± 122	271.3 ± 122	276.2 ± 106	201.5 ± 52
Ser *	124.5 ± 16.3	99.4 ± 23.5	131.8 ± 24.3	103.7 ± 19.5	129.3 ± 2~~87.4~~ [d]
Thr	120.5 ± 37.5	112 ± 0	128.2 ± 43.5	131.3 ± 45.1	142.7 ± 26.7
Trp	69.5 ± 1.6	59.2 ± 15.5	53.9 ± 11.1	65.4 ± 16.3	64.5 ± 12.3
Tyr **	85.5 ± 12.1	85.7 ± 11.7	80.2 ± 17	95.4 ± 28.8	66.1 ± 17 [d]
Val	290 ± 89.1	216.5 ± 29	203.3 ± 32.5	232.8 ± 57	213.5 ± 39.6
Acetylornithine	<LOQ	<LOQ	<LOQ	<LOQ	<LOQ
ADMA	0.6 ± 0.0	0.6 ± 0.3	0.4 ± 0.2	0.5 ± 0.1	0.4 ± 0.1
SDMA	<LOQ	<LOQ	<LOQ	<LOQ	<LOQ
α-Aminoadipic acid	<LOQ	<LOQ	<LOQ	<LOQ	<LOQ
Carnosine	<LOQ	<LOQ	<LOQ	<LOQ	<LOQ
Creatinine	107 ± 28.4	100.6 ± 4.8	76.6 ± 16.1	89.1 ± 31.5	75.9 ± 18.7
DOPA	<LOQ	<LOQ	<LOQ	<LOQ	<LOQ
Dopamine	<LOQ	<LOQ	<LOQ	<LOQ	<LOQ
Histamine	<LOQ	<LOQ	<LOQ	<LOQ	<LOQ
Kynurenine ***	4.3 ± 0.9 [b]	10.1 ± 6.5 [a,c,d]	3.7 ± 1.3 [b]	4.5 ± 1.6 [b]	3 ± 1 [b,d]
Methionine sulfoxide	<LOQ	<LOQ	<LOQ	<LOQ	<LOQ
Nitro-Tyr	<LOQ	<LOQ	<LOQ	<LOQ	<LOQ
Putrescine	0.1 ± 0	0.1 ± 0.0	0.2 ± 0.1	0.2 ± 0.1	0.1 ± 0.1
Serotonin	0.5 ± 0.2	0.2 ± 0.1	0.4 ± 0.2	0.5 ± 0.3	0.6 ± 0.3
Spermine	<LOQ	<LOQ	<LOQ	<LOQ	<LOQ
Cis- 4-OH Pro	<LOQ	<LOQ	<LOQ	<LOQ	<LOQ
Trans 4-OH Pro ***	16.1 ± 9.3	11.8 ± 1.7	12.4 ± 7	13.3 ± 8.1	3.2 ± 4.5 [d]
Taurine	83.1 ± 10.3	73.5 ± 19.6	114.7 ± 28.8	115.2 ± 55.1	104 ± 28.9
lysoPC a C14:0	<LOQ	<LOQ	<LOQ	<LOQ	<LOQ
lysoPC a C16:0 *	195 ± 96.2 [d]	118 ± 24	128.5 ± 14	120.2 ± 27~~6.9~~ [a]	141.6 ± 24.8
lysoPC a C16:1	6.4 ± 2.9	3.8 ± 2	5.1 ± 1.7	4.2 ± 1.5	4 ± 0.9
lysoPC a C17:0 **	4.6 ± 2.6 [b,c,d]	1.9 ± 0.3 [a]	1.9 ± 0.5 [a]	2.3 ± 0.7 [a]	2.6 ± 0.6 [a]
lysoPC a C18:0 **	61.9 ± 42~~1.4~~ [b,c,d]	28.8 ± 3.5 [a]	31.9 ± 4.5 [a]	31.6 ± 8.2 [a]	41.2 ± 11.4
lysoPC a C18:1 *	47.3 ± 26~~5.7~~ [c,d]	26.7 ± 6.6	33.9 ± 7.3 [a]	27.6 ± 6.9 [a]	30.6 ± 6.6 [a]
lysoPC a C18:2 *	71.8 ± 54 [d]	36.5 ± 2.9	48.9 ± 16.7	36.1 ± 9.3 [a]	44 ± 13.2
lysoPC a C20:3 *	5 ± 3.9 [b,d]	2.2 ± 0.6 [a]	3.1 ± 0.6	2.6 ± 0.9 [a]	2.8 ± 0.7

lysoPC a C20:4	11.5 ± 6.4	8 ± 0.3	10.3 ± 2.7	8.3 ± 1.7	8.6 ± 2.7
lysoPC a C24:0	0.9 ± 0.1	1.1 ± 0.1	0.9 ± 0.1	1.1 ± 0.5	0.7 ± 0.6
lysoPC a C26:0	1.9 ± 0.3	2.2 ± 0.1	1.8 ± 0.3	2.1 ± 0.7	1.5 ± 1.4
lysoPC a C26:1	0.9 ± 0	1.3 ± 0.1	1.1 ± 0.2	1.3 ± 0.4	0.9 ± 0.8
lysoPC a C28:0	1.2 ± 0.3	1.1 ± 0	1 ± 0.1	1.3 ± 0.4	1 ± 0.8
lysoPC a C28:1	1.2 ± 0.1	1.3 ± 0.1	1.1 ± 0.1	1.4 ± 0.4	1.1 ± 0.9
PC aa C24:0	0.7 ± 0.2	0.9 ± 0.1	0.7 ± 0.1	0.9 ± 0.3	0.6 ± 0.5
PC aa C26:0	4.3 ± 0.1	6.2 ± 0.4	4.5 ± 0.9	5.4 ± 1.8	3.3 ± 3.2
PC aa C28:1	4 ± 0.3	3.2 ± 0.7	2.8 ± 0.2	3.7 ± 0.7	3.9 ± 1.3
PC aa C30:0	4.6 ± 0.8	4.6 ± 2.6	3.8 ± 1.2	5.4 ± 2.2	5.2 ± 2
PC aa C30:2	0.2 ± 0.1	0.1 ± 0.2	0.2 ± 0.1	0.2 ± 0.1	0.4 ± 0.3
PC aa C32:0	12.8 ± 1.3	15.1 ± 6.4	12.6 ± 2.8	14.2 ± 3.6	13.8 ± 3.6
PC aa C32:1	16.8 ± 3.9	16.4 ± 15.8	17.5 ± 9.9	19.5 ± 11	16.2 ± 6.7
PC aa C32:2 *	2.6 ± 1.1	2.1 ± 1.1	1.8 ± 0.4	2.9 ± 1.1	4.0 ± 1.5 ^{c}
PC aa C32:3	0.4 ± 0.1	0.4 ± 0.1	0.3 ± 0	0.5 ± 0.1	0.5 ± 0.2
PC aa C34:1	192 ± 41	184.5 ± 112	163.8 ± 38	187.2 ± 50	205.5 ± 54
PC aa C34:2 **	303.5 ± 66	278.5 ± 79	253.8 ± 32	289.3 ± 50	361.7 ± 83 ^{c,d}
PC aa C34:3	13.7 ± 6.5	10 ± 6.9	9.4 ± 2.9	12.8 ± 5	15.1 ± 4.7
PC aa C34:4 **	1.2 ± 0.7	0.7 ± 0.4	0.7 ± 0.3	1.1 ± 0.4	1.6 ± 0.5 ^{c,d}
PC aa C36:0	1.5 ± 0.5	1.5 ± 0.9	1.5 ± 0.4	1.9 ± 0.8	2.4 ± 1
PC aa C36:1	48.3 ± 29.8	38 ± 23.3	33.1 ± 10.1	41 ± 11.7	45.6 ± 14.7
PC aa C36:2	205 ± 79.2	168.5 ± 51.6	151.8 ± 28.3	179.6 ± 34.7	226.9 ± 66.2
PC aa C36:3	108.1 ± 43.7	84.1 ± 39.5	85.4 ± 16.8	96.4 ± 24.5	120.8 ± 37.2
PC aa C36:4 **	129.1 ± 62.2	104.7 ± 30.2	114.9 ± 24.8	125.6 ± 23.2	167.7 ± 4~~65.7~~ ^{c,d}
PC aa C36:5	15.2 ± 5.6	17.9 ± 15.5	14.1 ± 4.3	20.7 ± 7.7	34.3 ± 21.8
PC aa C36:6 **	0.6 ± 0.2	0.6 ± 0.4	0.4 ± 0.1	0.7 ± 0.3	1.1 ± 0.5 ^{c,d}
PC aa C38:0	2.1 ± 0.2	2.7 ± 1.6	1.9 ± 0.3	2.4 ± 0.7	2.7 ± 1
PC aa C38:1	0.7 ± 0	0.9 ± 0.1	0.8 ± 0.2	1 ± 0.4	1.2 ± 0.6
PC aa C38:3	40.9 ± 21	28.4 ± 14.6	30.6 ± 13.8	37.2 ± 12.5	41.2 ± 13.8
PC aa C38:4	77.1 ± 40.9	54.3 ± 12.5	56.6 ± 16.3	66.6 ± 18.3	88.7 ± 30
PC aa C38:5 **	39.6 ± 17.7	32.3 ± 17.5	28.8 ± 6.3	35.6 ± 8.5	53.8 ± 1~~87.8~~ ^{c,d}
PC aa C38:6 **	54.7 ± 2.8	58 ± 36.2	47.8 ± 12.8	61.7 ± 16.3	84.6 ± 2~~98.3~~ ^{c,d}
PC aa C40:1	<LOQ	<LOQ	<LOQ	<LOQ	<LOQ
PC aa C40:2	0.3 ± 0	0.4 ± 0	0.3 ± 0	0.4 ± 0.1	0.4 ± 0.1
PC aa C40:3	0.4 ± 0.1	0.4 ± 0.1	0.4 ± 0	0.5 ± 0.1	0.5 ± 0.1
PC aa C40:4	3.4 ± 2.2	1.9 ± 0.7	2 ± 0.5	2.3 ± 0.6	2.7 ± 0.7
PC aa C40:5	9.1 ± 5.5	6 ± 3.9	5.2 ± 1.8	6.4 ± 2	8.2 ± 2.4
PC aa C40:6	24 ± 9.1	23.4 ± 18.4	17.5 ± 7.4	24.3 ± 7.9	28.1 ± 11.3
PC aa C42:0	0.4 ± 0	0.5 ± 0.2	0.4 ± 0.1	0.6 ± 0.2	0.5 ± 0.1
PC aa C42:1	0.3 ± 0.1	0.2 ± 0.1	0.2 ± 0	0.3 ± 0.1	0.3 ± 0
PC aa C42:2	0.2 ± 0	0.3 ± 0.1	0.2 ± 0	0.3 ± 0.1	0.2 ± 0.1
PC aa C42:4	0.2 ± 0	0.2 ± 0	0.1 ± 0	0.2 ± 0	0.2 ± 0
PC aa C42:5 **	0.3 ± 0.1	0.2 ± 0.1	0.2 ± 0	0.3 ± 0.1	0.4 ± 0.1 ^{c,d}
PC aa C42:6	0.4 ± 0.6	0.6 ± 0	0.6 ± 0	0.6 ± 0.2	0.7 ± 0.2
PC ae C30:0	0.6 ± 0	0.6 ± 0.2	0.4 ± 0.1	0.6 ± 0.2	0.5 ± 0.2
PC ae C30:1	0.8 ± 0	1 ± 0.1	0.8 ± 0.1	1 ± 0.3	0.8 ± 0.6

PC ae C30:2	0.2 ± 0	0.2 ± 0	0.2 ± 0	0.2 ± 0.1	0.2 ± 0.1
PC ae C32:1	2.4 ± 0	2.3 ± 0.7	2.4 ± 0.5	2.9 ± 1	2.8 ± 0.6
PC ae C32:2	0.7 ± 0	0.7 ± 0.1	0.7 ± 0.1	0.9 ± 0.2	0.9 ± 0.3
PC ae C34:0	1.3 ± 0.2	1.1 ± 0.5	0.9 ± 0.1	1.3 ± 0.5	1.4 ± 0.5
PC ae C34:1	9.1 ± 1	9.1 ± 4.8	7.3 ± 1.8	9.5 ± 3.7	9.4 ± 2.3
PC ae C34:2	8.8 ± 1.3	7.1 ± 2.5	7 ± 1.9	8.3 ± 3.2	10.3 ± 2.7
PC ae C34:3 **	3.9 ± 0.2	3.4 ± 0.6	5.3 ± 2	4.5 ± 2	6.6 ± 1.6 [c,d]
PC ae C36:0	0.7 ± 0	0.8 ± 0.4	0.8 ± 0.2	0.9 ± 0.3	0.8 ± 0.3
PC ae C36:1	8 ± 2	6 ± 2.4	5.2 ± 0.3	7.3 ± 2.5	8.2 ± 2.3
PC ae C36:2	13.2 ± 3.7	9.3 ± 2.2	8.6 ± 1.3	11.2 ± 3.5	13.3 ± 4
PC ae C36:3	5.8 ± 1.4	4.5 ± 1.5	4.8 ± 1.1	5.1 ± 1.8	6.4 ± 1.6
PC ae C36:4 *	11.9 ± 1.6	11.8 ± 4.4	9.3 ± 1.2	10.6 ± 3.2	13.6 ± 3.1 [d]
PC ae C36:5	6 ± 0.1	5.6 ± 2	7.3 ± 2	7.5 ± 2.6	9.6 ± 3
PC ae C38:0 **	1.4 ± 0.4	1.2 ± 0.6	1.1 ± 0.2	1.4 ± 0.4	2.2 ± 0.8 [c,d]
PC ae C38:1	0.5 ± 0.1	0.5 ± 0.2	0.6 ± 0.1	0.6 ± 0.2	0.6 ± 0.2
PC ae C38:2	2 ± 0.7	1.7 ± 0.3	1.6 ± 0.2	1.8 ± 0.5	2.1 ± 0.6
PC ae C38:3	3.7 ± 1.3	2.5 ± 0.9	2.5 ± 0.3	3.1 ± 0.9	3.7 ± 0.9
PC ae C38:4	10.6 ± 3.2	8.8 ± 1.2	7.5 ± 1.1	9 ± 2.5	10.6 ± 2.3
PC ae C38:5	12.5 ± 1.1	13.5 ± 4	11.4 ± 2.1	12.6 ± 3.1	14.6 ± 3.3
PC ae C38:6	4.7 ± 0.2	5.5 ± 3.4	4.5 ± 0.9	5.6 ± 2	7.2 ± 2.8
PC ae C40:1	1.7 ± 0.5	1.4 ± 0.2	1.4 ± 0.3	1.6 ± 0.3	1.6 ± 0.8
PC ae C40:2	1.4 ± 0.1	1.3 ± 0.5	1.3 ± 0.1	1.6 ± 0.4	1.9 ± 0.6
PC ae C40:3	0.8 ± 0.1	0.7 ± 0.1	0.8 ± 0.1	0.9 ± 0.2	1 ± 0.2
PC ae C40:4	1.8 ± 0.5	1.7 ± 0.4	1.6 ± 0.2	1.9 ± 0.5	1.9 ± 0.4
PC ae C40:5	2.9 ± 0.5	2.5 ± 1	2.3 ± 0.4	2.8 ± 0.8	3.1 ± 0.5
PC ae C40:6	3.8 ± 0.5	4 ± 2.2	2.7 ± 0.3	3.7 ± 1.1	4.4 ± 1.3
PC ae C42:0	<LOQ	<LOQ	<LOQ	<LOQ	<LOQ
PC ae C42:1	0.7 ± 0.1	0.7 ± 0	0.7 ± 0.1	0.8 ± 0.2	0.7 ± 0.4
PC ae C42:2	0.7 ± 0.1	0.6 ± 0.2	0.5 ± 0	0.7 ± 0.1	0.7 ± 0.3
PC ae C42:3	0.9 ± 0.1	0.8 ± 0.2	0.8 ± 0.1	1 ± 0.2	1 ± 0.4
PC ae C42:4	0.7 ± 0.1	0.6 ± 0.2	0.6 ± 0.1	0.8 ± 0.3	0.8 ± 0.2
PC ae C42:5	1.5 ± 0.1	1.5 ± 0.4	1.6 ± 0.4	1.8 ± 0.5	1.7 ± 0.3
PC ae C44:3	0.2 ± 0	0.2 ± 0.1	0.2 ± 0	0.3 ± 0.1	0.2 ± 0.1
PC ae C44:4	0.3 ± 0	0.3 ± 0.1	0.4 ± 0.1	0.4 ± 0.1	0.4 ± 0.1
PC ae C44:5	1.2 ± 0	1.2 ± 0.2	1.5 ± 0.6	1.7 ± 0.5	1.5 ± 0.4
PC ae C44:6	0.9 ± 0.1	0.9 ± 0.2	0.9 ± 0.3	1.1 ± 0.3	1 ± 0.2
SM(OH)C14:1 **	4.3 ± 0.7	2.6 ± 0.2	2.7 ± 0.6	3.8 ± 1.1	5.1 ± 1.4 [c,d]
SM(OH)C16:1 **	1.8 ± 0.2	1.3 ± 0.2	1.3 ± 0.2	1.8 ± 0.5	2.4 ± 0.7 [c,d]
SM(OH)C22:1 ***	5.9 ± 0.8	3.9 ± 0.1	4.7 ± 0.9	5.6 ± 1.6	8.7 ± 2.6 [b,c,d]
SM(OH)C22:2 ***	5.3 ± 0.6	3.5 ± 0	4.2 ± 1.1	5.4 ± 1.6	7.8 ± 2.3 [b,c,d]
SM(OH)C24:1 ***	0.6 ± 0	0.4 ± 0	0.6 ± 0	0.6 ± 0.2	0.9 ± 0.3 [b,c,d]
SM C16:0 **	58.5 ± 2.3	56.7 ± 1.1	61.3 ± 7.4	66.8 ± 12.5	81.5 ± 18.6 [d]
SM C16:1 **	7.5 ± 0.1	7.5 ± 0.2	7.3 ± 1.6	9.4 ± 2	11.6 ± 3.2 [c]
SM C18:0	10 ± 0.5	9.2 ± 1.7	11 ± 1.8	12.9 ± 2.9	16.4 ± 5.1
SM C18:1 **	4 ± 0.2	4.2 ± 1.2	4.2 ± 0.6	5.6 ± 1.5	7.1 ± 2.3 [c]
SM C20:2 ***	0.3 ± 0.1	0.2 ± 0	0.2 ± 0	0.3 ± 0.1	0.5 ± 0.1 [b,c,d]

SM C22:3 ***	1.1 ± 0.9	0.4 ± 0.3	0.8 ± 0.1	0.7 ± 0.3	2.5 ± 1.3 [b,c,d]
SM C24:0 ***	9 ± 3	6.7 ± 0.6	8.8 ± 0.5	9.4 ± 1.8	13.2 ± ~~43.2~~[b,c,d]
SM C24:1	22.8 ± 1.1	23.2 ± 0.1	30.2 ± 5.6	31.3 ± 6.7	35.5 ± 10.1
SM C26:0 ***	0.1 ± 0	0.1 ± 0	0.1 ± 0	0.1 ± 0	0.2 ± 0[a,b,c,d]
SM C26:1	0.2 ± 0	0.2 ± 0.1	0.3 ± 0.1	0.3 ± 0.1	0.4 ± 0.1
Hexoses	5028 ± 1561	6694 ± 2944	5928 ± 2387	4683 ± 1318	4109 ± 832

aa, two fatty acid residues bound to glycerol with ester bonds; AC, aceylcarnitine with fatty acid residue of given length; ADMA, asymmetric dimethylarginine; ae, one fatty acid residue bound to glycerol with ester bond, one with ether bond; PC, phosphatidylcholine with fatty acid residue of given length; SDMA, symmetric dimethylarginine; SM, sphingomyeline with fatty acid residue of given length; SM(OH), hydroxylated sphingomyeline with fatty acid residue of given length; *, ** and *** statistical significance in ANOVA with $p < 0.05$, $p < 0.01$ and $p < 0.001$, respectively. Statistical differences between groups in post-hoc analysis are indicated with [a,b,c,d].

Table S3. Comparison of the validation characteristics of the two best fitting hierarchical clustering of the patients with chronic obstructive pulmonary disease (COPD) (n = 25). In the principal component analysis (PCA) based clustering only the five highest principal components, which all explained >5% of total variance, were used. Sparse partial least squares discriminant analysis (sPLSDA) served as the positive control, since this clustering is performed on multivariate analysis that optimizes the dataset for predefined clusters (in the current analysis the Global Initiative for Chronic Obstructive Lung Disease (GOLD) A-D (2011) strata).

		**PCA**	**sPLSDA**
R package	Validation characteristic or description	COPD patients only<br>All data	COPD patients only<br>All data
	Optimal number of clusters	6	4
fpc	average distance between clusters	5.78	12.76
	average distance within clusters	2.79	3.34
	maximum cluster diameter	4.67	8.34
	minimum cluster separation	2.18	5.70
	average silhouette widths	0.36	0.68
	correlation between distances and means of same and different clusters	0.57	0.90
	ratio of minimum separation and maximum diameter	0.47	0.68
	ratio of minimum average dissimilarity between clusters and maximum average within cluster dissimilarity	1.15	2.45
	entropy of the distribution of cluster memberships	1.70	0.96
	ratio of average within and average between clusters	0.48	0.26
	Calinski-Harabasz pseudo F-statistic	13.33	49.18
cluster-Sim	Davies-Bouldin index	1.04	0.47
	Calinski-Harabasz pseudo F-statistic	10.38	46.29
	Baker and Hubert adaption of Goodman and Kruskal's gamma statistics	0.84	1.00
	Hubert and Levine internal cluster quality index	0.39	0.37
	Rousseeuw's Silhouette internal cluster quality index	0.32	0.68
clValid	average proportion of non-overlap	0.22	0.00
	average distance	2.92	2.87
	average distance between means	1.46	0.00
	Figure of merit	1.72	0.75
	Connectivity	27.07	12.10
	Dunn index	0.46	0.68
	Silhouette	0.33	0.68

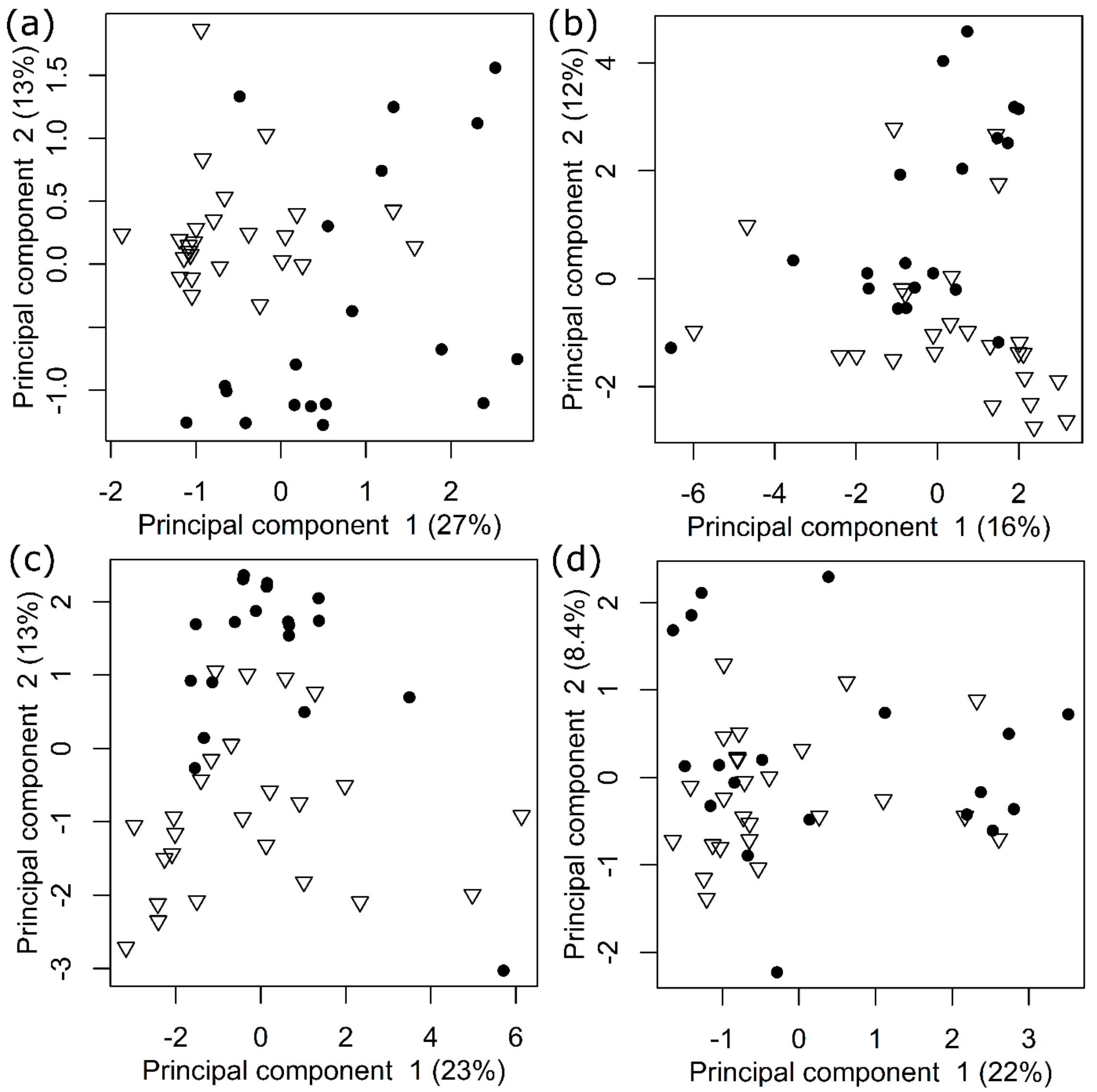

Figure S1. Principal component analysis of (a) targeted metabolites, (b) untargeted metabolic profiles, (c) untargeted metabolic profiles of blood serum only and (d) untargeted metabolic profiles of exhaled breath condensate only of the patients with chronic obstructive pulmonary disease (COPD, empty triangles) and the control individuals (solid circles). The percentage in parenthesis indicates the fraction from total variance explained by the respective principal component.

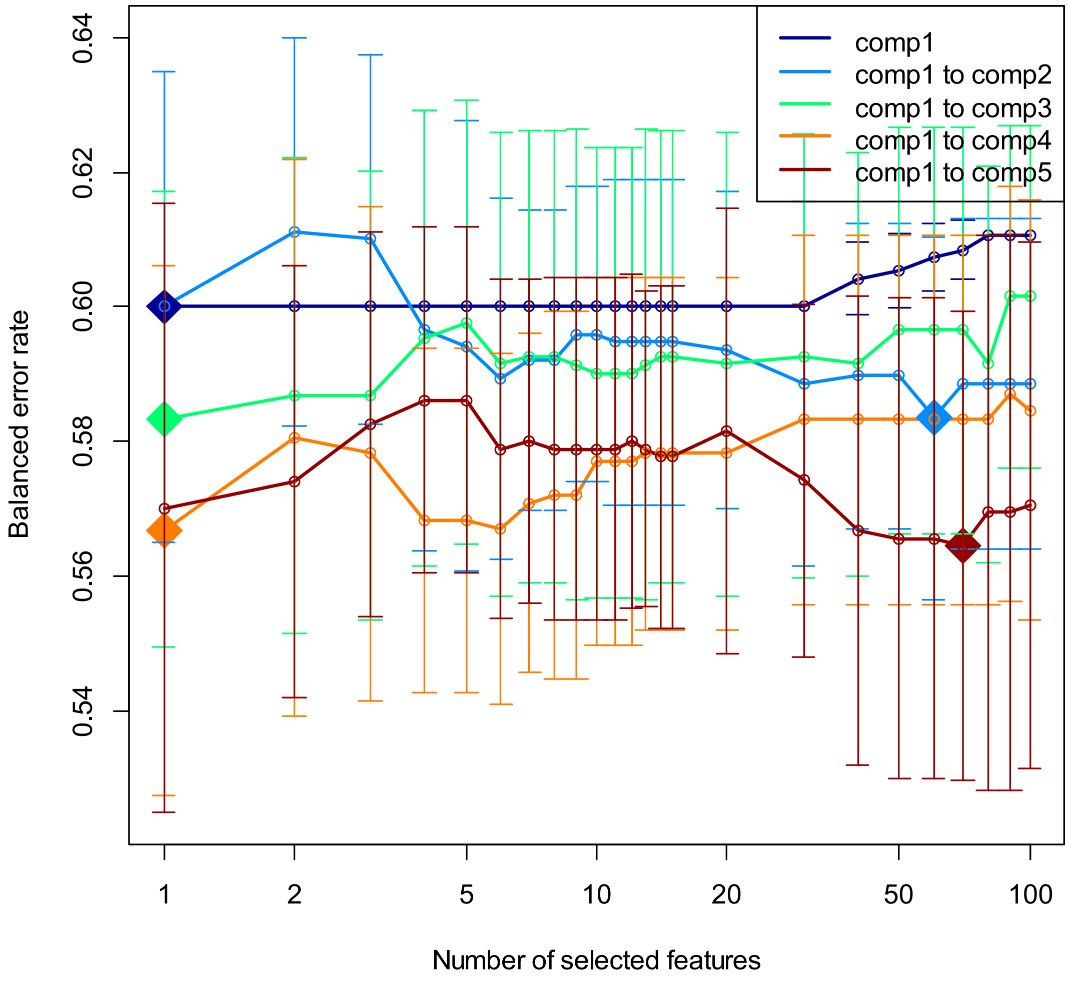

Figure S2. Tuning of sparse least squares discriminant analysis. Withing the error limits the performance of the model is relatively independent on number of components and number of parameters kept (x-axis). Two components (blue line) with 60 input variables was chosen for presentation in the article.
